# Supplementary material for: Resection Margin Status and Long-Term Outcomes after Pancreaticoduodenectomy for Ductal Adenocarcinoma: A Tertiary Referral Center Analysis
Source: Cancers (Basel). 2024 Jun 26;16(13):2347. doi: 10.3390/cancers16132347 (PMC11240367; doi:10.3390/cancers16132347)
Supplement: Supplementary file 1 [file cancers-16-02347-s001.zip › cancers-3055644-supplementary.pdf]

cancers

MDPI

Supplementary Materials: Resection Margin Status and Long-Term Outcomes after Pancreaticoduodenectomy for Ductal Adenocarcinoma: A Tertiary Referral Center Analysis

Giuseppe Quero, Davide De Sio, Claudio Fiorillo, Chiara Lucinato, Edoardo Panza, Beatrice Biffoni, Lodovica Langellotti, Vito Laterza, Giulia Scaglione, Flavia Taglioni, Giuseppe Massimiani, Roberta Menghi, Fausto Rosa, Teresa Mezza, Sergio Alfieri and Vincenzo Tondolo

Table S1. Site of margin positivity.

Margin positivity	R1 (*n*: 62)
**Retroportal lamina, *n* (%)**	15 (24.2)
**Pancreatic resection margin, *n* (%)**	2 (3.2)
**SMV root, *n* (%)**	36 (58.1)
**CBD, *n* (%)**	2 (3.2)
**Anterior surface, *n* (%)**	5 (8.1)
**Posterior surface, *n* (%)**	14 (22.6)
**Multiple R1 margins, *n* (%)**	10 (16.1)
